# Utilization of Antenatal Healthcare Services, Associated Factors, and Pregnancy Outcomes Among Postnatal Women Who Delivered in Selected Rural Healthcare Centers in Odisha, Southeastern India

**DOI:** 10.7759/cureus.78591

**Published:** 2025-02-05

**Authors:** Manulata, Dharitri Swain

**Affiliations:** 1 College of Nursing, All India Institute of Medical Sciences, Bhubaneswar, Bhubaneswar, IND; 2 Department of Obstetrics and Gynecological Nursing, All India Institute of Medical Sciences, Bhubaneswar, Bhubaneswar, IND

**Keywords:** antenatal care utilization, antenatal service utilization factors, barriers of anc utilization, birth outcomes, maternal healthcare

## Abstract

Background: Adverse pregnancy outcomes remain a prevalent health issue in India. Many of the negative consequences could be avoided if mothers used prenatal care. However, in many low-resource Indian situations, the use of this service and its drivers has not been thoroughly studied. Therefore, this study was conducted to assess the utilization of antenatal care (ANC) services and the various factors affecting this utilization and pregnancy outcomes.

Material and methods: A cohort study was conducted among 326 recently delivered mothers in Khordha District, Odisha, in the southeastern community of India. A multistage sampling technique was used to select the desired sample. Demographic variables were obtained through a structured demographic proforma; maternal knowledge was evaluated using a structured knowledge questionnaire; and a factor-related structured questionnaire was used to identify factors associated with ANC service utilization. An interview method was used to collect the information from the participants. The data were entered using EpiData version 1.4.4.0 (Buenos Aires, Argentina) and exported to IBM SPSS Statistics for Windows, Version 22 (Released 2013; IBM Corp., Armonk, New York) for analysis. Bivariate and multiple logistic regressions were applied to identify participants and predictor variables, respectively.

Results: This study revealed that 227 (69.6%) had attended four or more recommended antenatal visits. The main perceived barriers to ANC access were unplanned pregnancy, delay in pregnancy confirmation, uncooperative partner, lack of family support, and distance from the health facility. It was found that 287 (88.1%) of women had adequate knowledge about the danger signs of pregnancy, and 174 (53.4%) were aware of available ANC health services in their locality. Knowledge of women was revealed to be a major factor impacting the utilization of ANC health services in this study (crude odds ratio (cOR) 24.03; 95%CI 12.37-46.65; p<0.01). It is also found that poor antenatal utilization was associated with an increased likelihood of complications arising during the antenatal period (cOR 2.43; 95%CI 0.34-18.71, p=0.01), immediate postnatal complications (cOR 1.77; 95%CI 2.43 0.83-17.15; p=0.02), cesarean section and operative vaginal delivery rate (cOR 1.88; 95%CI 1.11-3.17; p=0.03), and post-term babies (cOR 5.64; 95% CI 1.31-24.4; p=0.01).

Conclusion: The utilization of WHO-recommended ANC service in the present study area was found to be poor. The quality of ANC and maternal awareness among the population were found to be the factors that influence women's good utilization of ANC. Underutilization of ANC health services had a significant impact on adverse pregnancy outcomes, such as complications that arise during the antenatal and immediate postnatal period, cesarean sections, and operative vaginal delivery rates, as well as post-term pregnancy. These results can be utilized to develop a health intervention program to enhance maternal health utilization practices and, ultimately, improve maternal and child health quality in low-resource settings in India.

## Introduction

The World Health Organization (WHO) anticipates that "every expecting mother and child receives the best possible treatment during antenatal, intrapartum, and the postpartum period." The primary way of achieving this goal is through well-utilized antenatal care (ANC), as the WHO has established a favorable link between ANC service utilization and birth outcomes [1]. The WHO estimates that 800 mothers pass away from avoidable causes associated with pregnancy and delivery daily, with 99% of all maternal mortality occurring in underdeveloped nations. Additionally, women living in rural regions, underdeveloped areas, and communities with low literacy rates have greater maternal death rates [2]. The WHO developed a new ANC model with fewer visits and recommendations for the evidence-based interventions to be given at each visit, including an antenatal assessment, screening for preeclampsia, HIV, anemia, and syphilis, and the use of preventive measures such as checking iron and folic acid dosage, anti-malarial precautions, immunization with tetanus (T.T.), and advice on danger signs of pregnancy [3]. The non-use of ANC services is strongly related to pregnancy and birth outcomes [4]. Prenatal healthcare service utilization is a severe public health concern, as poor usage of ANC significantly contributes to maternal mortality and morbidity worldwide, especially in developing nations [5,6].

The WHO recommended a new ANC model with at least eight scheduled visits for a positive pregnancy experience and a focused ANC model with at least four targeted prenatal checkups based on the advantages of ANC. Developing nations like India eventually embraced this recommendation [7]. Studies have shown that characteristics such as the woman's age, education, residence location, occupation, socioeconomic level, and religion impact the use of ANC [8].

In India, the National Health Mission (NHM) and Reproductive and Child Health (RCH) both seek to provide high-quality ANC, which includes a minimum of four ANC visits, weight and blood pressure checks, early registration, antenatal examinations, tetanus vaccination, hemoglobin tests, urine tests, consumption of IFA (iron and folic acid) tablets for 100 days, and anemia management [2,4,9]. Pregnancy problems such as anemia, preeclampsia, and eclampsia are more likely to occur in women who seek ANC infrequently during their antenatal period. Along with poor delivery outcomes such as preterm birth, low birth weight (LBW), and stillbirth, all of these variables also lead to increased maternal mortality [1].

Odisha has one of the highest maternal mortality rates (MMR) in India, with an MMR of 673 per 100,000 live births and more than 14,000 women dying annually from pregnancy-related complications. Odisha is one of the first Indian state governments to demonstrate a strong commitment to the Millennium Development Goals (MDGs), which were inspired by the United Nations (UN), by making one of the MDG objectives, maternal health, the focal point of its national development plan [10]. Various studies recommended that all pregnant women be counseled about the significance of ANC through antenatal counseling and birth preparedness classes, emphasizing the value of antenatal visits and the use of various ANC services for better birth outcomes [9,11-13]. Therefore, the present study aims to determine ANC utilization status, factors associated with the utilization, and related pregnancy outcomes.

## Materials and methods

Study design and setting

A community-based cross-sectional study was conducted to assess the utilization of ANC health services, associated factors, and pregnancy outcomes in the state of Odisha, southeastern India. The study was carried out for a six-month period after taking the authorized, ethical permission of community stakeholders. The study was conducted in primary health centers, community health centers, and the district headquarters hospitals in the two zones of Khordha District, Odisha, to assess the utilization of ANC health services, factors associated with it, and related pregnancy outcomes.

The district has a total population of 2,246,341, of which 28% are in the reproductive age group. Khordha is the most urbanized district of Odisha, having two subdivisions and 10 blocks [10]. There is a lack of data regarding women's utilization of ANC despite the district's longstanding intervention aimed at enhancing maternal health. Consequently, the study area was selected to fill this gap and gather information about the utilization of ANC health services, associated factors, and pregnancy outcomes.

Study population and sampling

The sample size was calculated using a single population formula (n = (Z1-α/2 )^2^ (p)(q)/d^2^) based on the following assumptions: using 74% prevalence of ANC utilization in Himachal Pradesh, India [14], 95% confidence interval (CI), 5% precision, and 10% non-response rate, which yielded the sample size to be 330.

A two-stage sampling technique was applied to select the study participants from the population of individuals who gave birth in the 12 months preceding the survey. In the first stage of the sampling technique, primary health centers (Mendhasala), community health centers (Tangi blocks), and district headquarters hospitals (Khordha) were selected randomly. Finally, the second stage of the sampling technique was accomplished by selecting the study participants through a systematic sampling technique from their respective health centers. The study participants were all women who delivered at the study centers within the study period from October 2023 to March 2024. Women who never received ANC, who had no antenatal records, and those who were diagnosed as mentally retarded/handicapped/mentally ill and unable to respond to the question were excluded from the study. Participants who satisfied the inclusion criteria were interviewed by the primary investigator.

Data collection instrument and procedure

Data were collected using an interviewer-administered structured questionnaire. The questionnaire was prepared in English, including all relevant variables based on the objectives of the study, and translated to the local language (Odia) for the interview. The tool included sociodemographic variables and factors of ANC health service utilization, which included maternal knowledge about ANC health services and the quality of maternal healthcare services received by the women. The quality of maternal healthcare services included prenatal, labor and delivery, and immediate postnatal services per the standard ANC protocol. The tool was prepared by reviewing different literature and guidelines. The content validity of the tool was established by seven experts from the fields of community medicine and obstetrics. Scale-Content Validity Item/Average (S-CVI/Ave=0.97) and Scale-Content Validity Item/Universal Agreement (S-CVI/UN=0.86) both had high values, indicating that the instrument's overall content validity index was appropriate. The test-retest approach was used to determine the tool's reliability, and the results showed that Pearson correlation (r) = 0.997. For participant comprehension, the tool was translated into an Odia regional language; language experts then retranslated the Odia tool into English for linguistic validity.

Ethical consideration

The study was conducted after obtaining permission from the Institutional Ethical Committee and the concerned authority of the study areas (CDMO, Khordha District). All the participants available in the settings were made comfortable by asking general questions about the baby and her health. After introducing herself, the researcher provided the woman with a thorough explanation of the study's goal and how long it would take to finish the surveys. The researcher discussed the participant information sheet in advance, which included information on voluntary participation in the study, refusal, or withdrawal by the subject at any time without any negative consequences for future hospital care. Data were collected after obtaining informed consent from the participants through both oral and written consent forms based on the literacy level of the participants. The average duration to complete all questionnaires by one participant was 15-20 minutes; the investigator interviewed each participant, and at the same time, cross-checking was done with all the antenatal records available with the participants and along with labor case book records. Finally, the senior investigator reviewed all collected forms for accuracy and completeness.

Data management and analysis

Data were coded and entered using EpiData version 1.4.4.0 (Buenos Aires, Argentina), and all analyses were done with IBM SPSS Statistics for Windows, Version 22 (Released 2013; IBM Corp., Armonk, New York). The study population was described using proportions and means (SD) according to explanatory variables and place of delivery. Logistic regression was used to determine the differences in the study population's use of ANC health services. A multivariable logistic regression model was utilized to identify the significant factors that influence the use of ANC health services. At p<0.05, all statistical tests were deemed significant.

## Results

Maternal characteristics of the study participants

A total of 330 postnatal women who gave birth within the past 12 months were assessed for eligibility, and 326 women participated, making a response rate of 98.78%. The current mean age of the study participants was 25.57 ± 4.36. Most were married 307 (94.2%), and the mean marriage age was 21.75 ± 3.58. The majority of them belong to rural areas; 140 (42.9%) had completed primary education qualifications, 298 (91.4%) were homemakers, and 225 (69%) belonged to low socioeconomic status (family monthly income ≤7,315). About maternal characteristics, most of the participants were multipara 172 (52.7%) and had unplanned pregnancies 298 (91.4%) (Tables 1, 2).

**Table 1:** Sociodemographic characteristics of antenatal women (n=326)

Variables	f	%
Age (in years) (Mean ± SD)	25.57 ± 4.36	-
Age at marriage (in years)	21.75 ± 3.58	-
Religion		
Hindu	309	94.8
Muslim	17	5.2
Place of residence		
Rural	286	87.7
Urban	40	12.3
Marital status of the mother		
Married	307	94.2
Divorced	5	1.5
Widowed	14	4.3
Mother’s education		
No formal education	19	5.8
Primary	140	42.9
Secondary	138	42.3
Graduation and above	29	8.9
Mothers occupation		
Housewife	298	91.4
Govt. employee	6	1.8
Private employee	10	3.1
Any other	12	3.7
Husband’s education		
No formal education	10	3.1
Primary	125	38.3
Secondary	154	47.2
Graduation and above	37	11.3
Husband’s occupation		
Govt. employee	13	4.0
Private employee	47	14.4
Any other	266	81.6
Socioeconomic class		
Above poverty line	101	31.0
Below poverty line	225	69.0
Type of family		
Joint family	199	61.0
Nuclear family	127	39.0
Approximate distance from the healthcare center		
<5 km	68	20.9
>5 km	258	79.1
Received information through mass media	239	73.3

**Table 2:** Obstetrics information of women (n=326)

Variables	f	%
Parity		
0 (Nullipara)	6	1.84
1 (Prime para)	148	45.3
>1 (Multipara)	172	52.7
Abortion		
0	320	98.1
1	6	1.84
>1	0	0
Living children		
0	09	2.76
1	148	45.3
>1	169	51.8
Complications during past pregnancy		
Yes	39	12.0
No	287	88.0
Current pregnancy status		
Planned	28	8.6
Unplanned	298	91.4

ANC health service utilization and perceived barriers

Concerning the WHO recommendation of an ANC visit, it is recommended that at least four ANC visits are needed for a pregnant mother until delivery. In this study, most participants, 269 (82.51%), visited a health facility for ANC service at least once, whereas only 227 (69.60%) had at least four ANC visits for their index child. The majority, 247 (75.76%), of the study participants used the local primary health center for their ANC visit, followed by a health post and hospital. The main perceived barriers to ANC access were unplanned pregnancy, 51 (51.51%), delay in pregnancy confirmation, 42 (42.42%), uncooperative partner, 2 (12.12%), and lack of family support, 10 (10.10%).

Level of maternal knowledge about ANC and quality of maternal healthcare

Overall, more than half of the study participants, 197 (58.6%), had adequate knowledge regarding ANC. Among them, 125 (38.6%) had an understanding of ANC components and their benefits on maternal and newborn health; 174 (53.4%) participants had information about ANC services available in their locality; and 287 (88.1%) participants had inadequate knowledge of the danger signs of pregnancy.

Table 3 presents the quality of maternal healthcare services received by the women. With regard to good antenatal services, 203 (89.4%) of them registered in the first three months of pregnancy; all of them attended antenatal checkups in the health center and confirmed pregnancy by either a urinary pregnancy test or USG. The percentage of routine ANC service by pregnant mothers is comparatively better among adequate ANC utilizers.

**Table 3:** Quality maternal services received by the antenatal women USG: ultrasound; ASHA/AWW/ANM: accredited social health activist/Anganwadi worker/auxiliary nurse midwife

Variables	<4 Visits		≥4 Visits	
	f (n=99)	%	f (n=227)	%
Antenatal registration				
Pregnancy registered in health center in first three months of pregnancy	44	44.44	203	89.42
Attended antenatal checkups in the health center	98	98.98	227	100
Pregnancy confirmed by pregnancy test/USG	99	100	227	100
Antenatal card provided	99	100	227	100
Antenatal assessment				
Weight measurement was done	99	100	227	100
Blood pressure checked	99	100	227	100
Abdominal examination done by healthcare workers	99	100	227	100
Contacted with any healthcare workers (ASHA/AWW/ANM)	92	92.92	222	97.79
Antenatal advice received during pregnancy (diet, exercise, sleep, medication, etc.)	68	68.68	151	66.51
Vaccination and medication received				
Folic acid supplements are taken during the first three months of pregnancy	99	100	227	100
Iron supplements taken (at least 180 tablets)	81	81.81	206	90.74
Calcium supplements taken throughout pregnancy	94	94.94	223	98.23
Dewormed tablet (albendazole) taken	40	40.40	73	32.15
Received two doses of tetanus-diphtheria (Td) vaccines	98	98.98	222	97.79
Received anti-D immunoglobulin (in Rh-negative pregnancy)	04	100	14	100
Preparedness for delivery				
Decided place for delivery in advance	7	7.07	33	14.53
Arranged transport in advance	7	7.07	23	10.53
Intrapartum care services				
Had institutional delivery	92	92.92	220	96.91
Baby delivered by trained healthcare workers	92	92.92	218	96.03
Immediate postnatal care services				
Duration of stay in the facility after delivery (48 hours)	84	84.84	217	95.59
Early initiation of breastfeeding	21	21.21	64	28.19
Vaginal pads are provided for bleeding vaginally	94	94.94	221	97.35
Blood pressure monitored immediately after delivery	67	67.67	176	77.53
Postnatal advice				
Continuation of iron and calcium supplements	89	89.89	212	93.39
Information about follow-up	77	77.77	183	80.61
Exclusive breastfeeding for six months	49	49.99	141	62.11
Contraception advised	28	28.28	75	33.03

Factors associated with the utilization of ANC services

In this study, the results of the chi-squared test analysis showed that women who were less educated, with unemployed partners, living in low socioeconomic conditions, living further from the health center, and having unplanned pregnancies were independently associated with poor ANC service utilization (p<0.05). Further multivariable logistic regression analysis was performed among the factors found to be associated with ANC utilization. Through multivariable logistic regression, poor ANC health service utilization was associated with women having poor knowledge about ANC and not being aware of the danger signs of pregnancy. Women who were knowledgeable about ANC had 24 times higher odds of service utilization compared to those not having formal education (OR: 24.03; 95% CI 12.37-46.65; p<0.01). Women with adequate ANC utilization received good-quality maternal healthcare services until delivery (cOR 11.19; 95% CI 4.66-26.86; p=0.002) compared to women with poor antenatal utilization (Table 4).

**Table 4:** Factors associated with utilization of ANC health services (n=326) *Significant (p-value < 0.05) CI: confidence interval; cOR: crude odds ratio; ANC: antenatal care

Variables	Utilization of ANC		cOR and 95% CI	p-value
	<4 visits (n=99)	≥4 visits (n=227)		
Age (in years)				
<20	14	24	1	-
21-25	39	97	0.65 (0.25-1.69)	0.37
25-30	35	77	0.94 (0.42-2.07)	0.88
>30	11	29	0.83 (0.37-1.85)	0.65
Religion				
Hindu	96	213	1	-
Muslim	3	14	2.10 (0.59-7.49)	0.25
Place of residence				
Rural	85	201	1	-
Urban	14	26	0.78 (0.39-1.58)	0.49
Mother’s education				
No formal education	6	13	1	-
Primary	39	101	1.19 (0.42-3.37)	0.73
Secondary	48	90	0.86 (0.30-2.42)	0.78
Graduation and above	6	23	1.76 (0.47-6.62)	0.39
Mothers occupation				
Housewife	92	206	1	-
Govt. employee	1	5	2.23 (0.25-19.3)	0.46
Private employee	2	8	1.78 (0.37-8.58)	0.46
Any other	4	8	0.89 (0.26-3.04)	0.85
Husband’s education				
No formal education	4	6	1	-
Primary	31	94	2.02 (0.53-7.63)	0.29
Secondary	56	98	1.17 (0.31-4.31)	0.81
Graduation and above	8	29	2.42 (0.54-10.7)	0.24
Husband’s occupation				
Govt. employee	5	8	1	-
Private employee	13	34	1.63 (0.45-5.92)	1.63
Any other	81	185	1.43 (0.45-4.50)	1.43
Socioeconomic class				
Above poverty line	29	72	1	-
Below poverty line	70	155	0.89 (0.53-1.49)	0.66
Type of family				
Joint family	55	144	1	-
Nuclear family	44	83	0.72 (0.44-1.16)	0.18
Approximate distance from healthcare center				
<5 km	25	43	1	-
>5 km	74	184	1.45 (0.82-2.54)	0.19
Mass media information received				
Yes	76	163	1	-
No	23	64	1.30 (0.74-2.25)	0.35
Marital status of the mother				
Divorced	0	5	1	-
Married	93	214	-	-
Widowed	6	8	-	-
Age at marriage (in years)				
<20	47	99	1	-
21-25	40	97	1.05 (0.18-5.95)	0.95
26-30	10	27	1.21 (0.21-6.88)	0.82
>30	2	4	1.35 (0.21-8.55)	0.75
Parity				
0	3	3	0.42 (0.08-2.16)	0.30
1	48	100	0.89 (0.58-1.35)	0.59
>1	48	124	1	-
Abortion				
0	96	224	1	-
1	3	3	0.42 (0.08-2.16)	0.30
>1	0	0	-	-
Living				
0	5	4	0.34 (0.09-1.30)	0.11
1	48	100	0.89 (0.58-1.35)	0.59
>1	46	123	1	-
Complications during past pregnancy				
Yes	14	25	1	-
No	85	202	1.33 (0.66-2.68)	0.42
Current pregnancy status				
Planned	6	22	1	-
Unplanned	93	205	0.60 (0.23-1.53)	0.28
Knowledge of women about utilization of antenatal healthcare services				
Adequate	13	178	1	-
Inadequate	86	49	24.0 (12.37-46.65)	0.01*
Quality of antenatal care services				
Good	73	220	1	-
Average	26	7	11.19 (4.66-26.86)	0.002*

Relationship between ANC service utilization and maternal and fetal outcomes

Women who had less ANC utilization had encountered more than two times higher rate of pregnancy complications (cOR 2.43; 95%CI 0.34-18.71, p=0.01) and a higher rate of developing immediate postnatal complications (cOR 1.77; 95%CI 2.43 0.83-17.15; p=0.02). Women with poor ANC utilization had five times higher odds of giving birth to post-term babies (cOR 5.64; 95%CI 1.31-24.4; p=0.01) and a higher rate of undergoing cesarean delivery or operative vaginal delivery (cOR 1.88; 95%CI 1.11-3.17; p=0.03) (Table 5).

**Table 5:** Relationship between ANC service utilization and maternal and fetal outcomes *Significant (p-value < 0.05) CI: confidence interval, cOR: crude odds ratio; IUFD: intrauterine fetal demise; ANC: antenatal care; LSCS: lower segment cesarean section

Variables	Utilization of ANC		cOR (95% CI)	p-value
	<4 visits f(%)	≥4 visits f(%)		
Maternal and fetal outcomes				
Complications during the antenatal period	32 (32.32)	9 (9.09)	2.43 (0.34-18.71)	0.02*
Complications during the immediate postnatal period	22 (22.22)	11 (4.84)	1.77 (0.83-17.15)	0.03*
Stillbirth/IUFD	2 (2.02)	1(0.44)	0.87 (0.07-9.72)	0.91
Spontaneous abortion	3 (3.03)	3(1.3)	0.42 (0.08-2.16)	0.30
Neonatal complication	3 (3.03)	3(1.3)	0.42 (0.08-2.16)	0.30
Mode of delivery (LSCS)	26 (27.2)	92 (40.5)	1.88 (1.11-3.17)	0.02*
Preterm	7 (7.07)	7 (3.1)	2.43 (0.83-7.15)	0.10
Post-term	24 (10.5)	2 (2.02)	5.64 (1.31-24.4)	0.01*

## Discussion

Understanding the demographic characteristics associated with ANC utilization can help identify disparities and inform targeted interventions to improve access and utilization of ANC services among specific populations. It is important to note that these characteristics can interact and vary across different contexts, so local factors should also be taken into account when analyzing ANC utilization. Numerous studies revealed a wide range of sociodemographic factors associated with ANC health service utilization. Ethnicity, women's education and employment, the ability of women to make decisions, and health system variables were the key variables determining ANC use [5,12,14-16]. It was also reported that failing to complete the recommended visits was connected with women who were less educated, living in lower-income circumstances, having higher birth orders, living distant from the health center, and living in rural areas [17-19]. Other studies found that mothers of younger ages, husbands with formal education, prior early ANC visits, and those who received four or more ANC visits in previous pregnancies were significantly associated with adequate ANC utilization [8,20,21]. The present study also identified that similar sociodemographic variables, such as women who were less educated, with unemployed partners, living in low socioeconomic conditions, more distant from the health center, and with unplanned pregnancies, were independently associated with poor ANC service utilization.

In the present study, it was reported that only 69.60% had at least four ANC visits, and the reasons for not utilizing at least four antenatal visits were mainly lack of knowledge, the pregnancy was unplanned, there was a delay in recognizing that one was pregnant, lack of family support, the partner has not accepted the pregnancy, and distance to a health facility. Similar findings are also reflected in other literature where utilization of at least four ANC visits was found to be poor, and the main perceived barriers to ANC access were a lack of awareness of the service and distance to a health facility [18,19].

The ANC utilization in terms of the number of antenatal visits was reported in many Indian and non-Indian studies. Different studies reported different levels of antenatal visits. A study found that 61.7% of mothers in Eastern Ethiopia had fewer than four visits [22]. Similarly, in a study conducted in Ghana, 69.0% reported utilizing at least four or more ANC services [23]. In another study of pregnant women attending antenatal clinics at tertiary care hospitals in Maharashtra, India, it was discovered that approximately 70% of the women adequately received ANC, and there was a statistically significant correlation between ANC knowledge and practice [24]. Another study conducted in Macro, USA, found that 63% of women showed higher than recommended ANC utilization; 52% had less than 80% of recommended routine care [25]. Our study found that 69.60% had at least four antenatal visits, 75.7% attended antenatal checkups within the first trimester, and 95.7% had institutional delivery. Women with adequate antenatal visits received good-quality ANC services.

ANC health service utilization plays a crucial role in promoting positive pregnancy outcomes. Regular and appropriate ANC can help detect and manage potential complications, ensure the health and well-being of both the mother and the fetus, and improve overall pregnancy outcomes. According to a systematic review and meta-analysis, sufficient ANC utilization was linked to a 20% decrease in infant deaths and a 16% decrease in stillbirths. Some previous research also discovered that proper ANC utilization was linked to a lower risk of preterm delivery, low birth weight, and stillbirth [14,26-28]. Another study showed that women who got sufficient ANC were less likely to experience obstetric difficulties following birth, such as postpartum hemorrhage and protracted labor. Additionally, the study discovered that women who got proper ANC were more likely to give birth in a hospital, which can facilitate access to emergency obstetric treatment in the event of problems [29]. Our study also proved a significant association between poor ANC utilization and adverse pregnancy outcomes, such as complications during the antenatal and immediate postnatal period, cesarean mode of delivery, and post-term pregnancy.

The present study adopted a cohort observational study design and included a good number of samples for sample representation of the selected district, Odisha state, which adds to the strength of the study. As it was conducted in the southeastern part of India, which may limit the generalizability of the study findings at the national level, the representative sample was taken from the district. Recall bias may be introduced due to the reliance on self-reported information from study participants in the survey, but most of the antenatal data was identified by assessing all the antenatal records. The knowledge questionnaire might be subjected to information contamination due to assistance from family members present with the mothers during their responses. This was controlled by conducting face-to-face interviews with individual participants to respond to the questionnaire independently without taking help from any information sources. During data collection, the presence of courtesy and social disability bias was evident due to the researcher wearing a clinical uniform.

## Conclusions

Although the majority of participants had an adequate level of knowledge of ANC health services, the utilization of standard ANC health services was poor, as all pregnant women had not attended at least four recommended antenatal visits. The present study identified the quality of ANC service and maternal knowledge regarding ANC services and the danger signs of pregnancy as two influencing factors for good utilization of ANC by pregnant women. It is also found that poor ANC utilization was associated with an increased likelihood of complications arising during the antenatal and immediate postnatal period, higher rates of cesarean delivery, as well as post-term deliveries as some poor pregnancy outcomes. These results can be utilized to develop a health intervention program with preconception counseling to enhance maternal health utilization practices and, ultimately, help improve pregnancy outcomes.
